# Outcome of life-threatening malaria in African children requiring endotracheal intubation

**DOI:** 10.1186/1475-2875-6-51

**Published:** 2007-04-30

**Authors:** Patrick Gérardin, Christophe Rogier, Amadou S Ka, Philippe Jouvencel, Bakary Diatta, Patrick Imbert

**Affiliations:** 1 Department of Paediatrics, Hôpital Principal, Dakar, Senegal; 2 Neonatal and Paediatric Intensive Care Unit, Pôle Mère-Enfant, Groupe Hospitalier Sud-Réunion, Saint-Pierre, La Réunion Island, France; 3 Research Unit in Parasitological Biology and Epidemiology, Institut de Médecine Tropicale du Service de Santé des Armées – IFR 48, Le Pharo, Marseille, France; 4 Department of Neonatology and Paediatrics, Centre Hospitalier de la Côte Basque, Bayonne, France; 5 Intensive Care Unit, Hôpital Principal, Dakar, Senegal; 6 Department of Infectious Diseases and Tropical Medicine, Hôpital d'Instruction des Armées Bégin, 69 avenue de Paris, 94160 Saint-Mandé, France

## Abstract

**Background:**

Little is known about children undergoing critical care for malaria. The purpose of this survey was to evaluate the outcome in African children requiring endotracheal intubation for life-threatening malaria.

**Methods:**

All children with a primary diagnosis of severe malaria (2000 WHO definition) requiring endotracheal intubation, hospitalised over a five-year period, within a tertiary-care hospital in Dakar, Senegal, were enrolled in a retrospective cohort study.

**Results:**

83 consecutive patients were included (median PRISM h_24 _score: 14; IQR: 10–19, multiple organ dysfunctions: 91.5%). The median duration of ventilation was 36 hrs (IQR: 4–72). Indications for intubation were deep coma (Glasgow score ≤7, n = 16), overt cortical or diencephalic injury, i.e, status epilepticus/decorticate posturing (n = 20), severe brainstem involvement, i.e., decerebrate posturing/opisthotonus (n = 15), shock (n = 15), cardiac arrest (n = 13) or acute lung injury (ALI) (PaO_2_/FiO_2 _<300 Torr, n = 4). Death occurred in 50 cases (case fatality rate (CFR), 60%) and was associated with multiple organ dysfunctions (median PELOD_h24 _scores: 12.5 among non-survivors *versus *11 among survivors, p = 0.02). Median PRISM_h24 _score was significantly lower when testing deep coma against other indications (10 *vs *15, p < 0.001), ditto for PELOD_h24 _score (2.5 *vs *13, p = 0.02). Multivariate analysis identified deep coma as having a better outcome than other indications (CFR, 12.5% *vs *40.0 to 93.3%, p < 0.0001). Decerebrate posturing/opisthotonus (CFR 73.3%, adjusted relative risk (aRR) 10.7, 95% CI 2.3–49.5) were associated with a far worse prognosis than status epilepticus/decorticate posturing (CFR 40.0%, aRR 5.7, 95% CI 1.2–27.1). Thrombocytopaenia (platelet counts <100,000/mm^3^) was associated with death (aRR 2.6, 95% CI 1.2–5.8) and second-line anticonvulsant use (clonazepam or thiopental) with survival (aRR 0.4, 95% CI 0.2–0.9). Complications, mostly nosocomial infections (n = 20), ALI/ARDS (n = 9) or sub-glottic stenosis (n = 3), had no significant prognostic value.

**Conclusion:**

In this study, the outcome of children requiring intubation for malaria depends more on clinical presentation and progression towards organ failures than on critical care complications *per se*. In sub-Saharan Africa, mechanical ventilation for life-threatening childhood malaria is feasible, but seems unlikely to dramatically improve the prognosis.

## Background

Malaria remains one of the most serious infections on a global basis. In Africa it accounts for about 20% of mortality in children under five [1]. Severe falciparum malaria generally has a poor prognosis with a 10–30% case fatality rate, due mainly to the three most frequent clinical forms of severe childhood malaria: severe malarial anaemia (SMA), cerebral malaria (CM) and respiratory distress syndrome (RDS) [2-8]. The availability of blood transfusions improves the survival of SMA [4,5,8], whereas the prognosis for CM, prevalent in hypoendemic areas, is still poor in the absence of resuscitation capability. CM has become the most serious malaria-threat in urban settings [3,4,9]. According to recent estimates, the yearly burden of CM is about 575,000 cases, 110,000 deaths (case fatality rate, 19.1%), and between 60 to 100,000 children discharged with neurological sequelae. The toll of long-term disabilities such as learning or cognitive deficits may be more widespread [8,10,11].

Diffuse or focal brain ischaemia, ring haemorrhages and transtentorial herniation are common patterns of the central nervous system involvement (CNS) in fatal CM. However, cerebral oedema was thought to be a non-specific agonic event [7]. In recent years, shock/hypovolaemia and metabolic acidosis have been stressed to be the leading causes for death in children, irrespective of the clinical form [12,13].

In sub-Saharan Africa, most hospitals face the curse of malaria without access to paediatric intensive cares. Severe forms of malaria are rare among expatriate children, and critical care for childhood malaria is not well-documented in developed countries. Thus, outcomes in children intubated for life-threatening malaria have not been assessed. In non-immune adults mechanically-ventilated for severe malaria, high cases fatality rates (25 to 75%) were observed both for residents of endemic countries [14,15] and travelers returning from malarial areas [16,17]. However, it is not clear whether critically-ill patients with malaria die from malaria or critical care complications.

The aim of the current study was to investigate the outcome and complications in African children requiring endotracheal intubation during resuscitation for life-threatening malaria.

## Methods

### Study design and setting

This retrospective observational cohort study took place in the Hôpital Principal, a tertiary-care hospital in Dakar, Senegal. All children who required endotracheal intubation for life-threatening falciparum malaria between January 1, 1996 and December 31, 2000, either in the 15-bed Intensive Care Unit (ICU), or in one of the two emergency rooms on the paediatric ward were evaluated. Whatever the ward, the mean ratios nurses: patients were 1:5 on day or 1:10 on night.

### Data collection

The variables of interest, recorded during the first 24 hrs of hospitalisation, included age, gender, duration of coma prior to admission, previous anti-malarial treatment, the 2000 World Health Organization (WHO) criteria for severe malaria (SM)[6], nutritional status [18], coexisting diseases, community-acquired infections, the Paediatric Risk of Mortality (PRISM_h24_) score [19], and organ dysfunctions, as assessed by the Paediatric Logistic Organ Dysfunction (PELOD_h24_) score [20]. PRISM_h24 _and PELOD_h24 _were assessed from full medical records and bedside daily boards enabling reliable recalculations of their values [19,20]. Consciousness was assessed using the Blantyre coma scale (BCS, range: 0–5) [21], and the revised Glasgow coma scale (GCS, range: 3–15) [22].

Blood gases were performed at admission in cases of clinically evident respiratory distress, *e.g.*, sustained low chest wall recession, deep acidotic breathing or Küssmaul ventilation, or around intubation. Lactataemia was not available.

Indications for intubation included: (1) *deep coma *(GCS ≤ 7 or BCS ≤ 2, but no status epilepticus or abnormal posturing); (2) coma with overt cortical/diencephalic injury as witnessed by *persistent status epilepticus or flexor (decorticate) posturing*; (3) coma with brainstem involvement as witnessed by *extensor or opisthotonic (decerebrate) posturing*; (4) *severe respiratory distress*, defined as an acute lung injury (ALI) with pulmonary infiltrates and a PaO2/Fio2 < 300 Torr [40.1 kPa]; (5) shock, defined as systolic blood pressure <50 (under 5 years) or <80 (beyond 5) mmHg; (6) *cardiac arrest*.

Neurological sequelae [23-26] were assessed on discharge and on follow-up. They were categorised using the Paediatric Cerebral Performance Category (PCPC) scale which distinguishes six levels of CNS disorders: (1) *normal*; (2) *mild disability*; (3) *moderate disability*; (4) *severe disability*; (5) *coma *or *vegetative state*; (6) *brain death *[27].

### Case management

Endotracheal intubations were performed by intensivists in the ICU and by paediatricians (intensive care trained) in the paediatric *emergency *rooms. The ICU respirators were Servo 900^® ^(Siemens, Munich, Germany) whilst in the paediatric emergency rooms, either a Babylog HF^® ^(Dräger, Lübeck, Germany) or two Osiris^® ^(Taema, Anthony, France) were used, according to their availability. Tuning of ventilation parameters was left to the physicians. However, except for acute respiratory distress syndrome (ARDS), parameters displayed physiological ranges: usual pressures for child alveolar recruitment (P_peak_: 15–25 cmH_2_0, PEEP: < 5 cmH_2_0), normal tidal volumes (V_T_: 6–8 ml/kg body weight), respiratory rates (20–60 breaths *per *min) and sufficient FiO2 to obtain a spO2 of up to 92%. Children with ARDS were ventilated with low tidal volumes and high PEEP.

Children with SM were treated according to WHO guidelines [6]. The impossibility of determining previous anti-malarial treatments frequently restricted the use of quinine loading doses. Quinine formate (10 mg/kg salt, *e.g.*, 8.3 mg/kg base) was infused intravenously every 8 hrs for 3–7 d, followed by oral chloroquine (25 mg/kg for 3 d).

Blood transfusions for SMA (Hgb <5 g/dL) were restricted to children with clinically evident respiratory distress, as defined above. Shock was treated with vascular expansion (colloids or crystalloids) adding vasoactive drugs (dopamine, dobutamine) if necessary.

Hypoglycaemia (<2.2 mmol/L) was treated with a slow IV 30% glucose followed by dextrose 5% infusion. Acidosis was treated with sodium bicarbonate only in case of cardiac arrest. After rehydration and parenteral furosemide, severe persistent renal failure was treated with haemodialysis.

A single convulsion was treated by diazepam (slow IV 0.03 mg/kg or intrarectal 0.05 mg/kg). A loading dose of 5–15 mg/kg phenobarbital was given by slow venous infusion for CM or multiple convulsions (> 1/24 hrs). If seizures persisted clinically, second line anticonvulsivants were infused, using increasing doses of clonazepam, combined with thiopental if clonazepam failed.

Intra-cranial hypertension (protracted episodes of decorticate or decerebrate rigidity in addition to dilated sluggish pupils) was treated with 10–20 ml/kg of 20% mannitol and short-time induced hypocapnia (PCO_2 _adjusted to 25–35 Torr [3.3–4.7 kPa]).

Parenteral antibiotics were limited to treat concomitant community-acquired and nosocomial infections, defined if occurring after 48 hrs of stay.

### Statistical analysis

The indications for endotracheal intubation were analysed according to malaria severity and according to organ dysfunctions, respectively in comparing medians of PRISM and PELOD scores with Kruskal-Wallis or Mann-Whitney tests, as appropriate. Primary endpoint was outcome, survival or death, and secondary endpoints were complications and neurological status, evaluated at discharge.

Kaplan-Meier curves were drawn to compare survival relative to the indications for intubation. A Cox proportional hazards regression model was performed to determine independent predictors of outcome. The cumulative incidence of nosocomial infections was graphically presented according to the duration of ventilation. All these analyses were computed in Stata^® ^(Stata Statistical Software: release 7; StataCorp. 2001). A *p *value < 0.05 was considered statistically significant.

## Results

### Population characteristics

During the study period, 502 children were hospitalized with a primary diagnosis of severe malaria. 83 of whom required mechanical ventilation (median duration: 36 hrs, IQR: 4–72). The median age was 8.6 years (IQR: 7–11), the sex ratio (male: female = 51:32) 1.6. At admission, median PRISM_h24 _score was 14 (IQR: 10–19) and multiple organ dysfunctions (MOD) were present in 91.5% (n = 76). During stay, the case fatality rate was 60.2% (n = 50). The population characteristics according to the status at discharge are presented in Table 1.

**Table 1:** Characteristics at admission of 83 children requiring endotracheal intubation for severe *Plasmodium falciparum *malaria in Dakar, Senegal, according to vital status

Parameter	Survivors (n = 33)	Non Survivors (n = 50)	P value
Male	22 (66.6)	29 (58.0)	0.49
Median age, years	9 (7 – 12)	9 (5 – 12)	0.70
Median time to admission, days	2 (1 – 3)	3 (2 – 5)	0.03
Duration of coma before intubation, hrs	13 (6 – 28)	11 (1 – 27)	0.21
Previous antimalaric treatment	22 (66.6)	28 (56.0)	0.37
PRISM_h24_	11 (6 – 18)	15 (11 – 20)	0.05
Number of Organ dysfunctions	2 (2 – 3)	2 (2 – 3)	0.24
PELOD_h24_	11 (2 – 21)	12. 5 (11 – 22)	0.02
Blantyre Coma Score	3 (1 – 3)	2 (3 – 4)	0.05
Glasgow Coma Score	8 (7 – 9)	7 (9 – 11)	0.03
Highest temperature, °C	39.0 (38.6 – 39.8)	39.0 (37.9 – 40.0)	0.32
Heart pulse rate,/min	132 (120 – 151)	136 (116 – 160)	0.48
Respiratory rate,/min	35 (25 – 46)	44 (36 – 52)	0.09
Mean arterial blood pressure, mmHg	73 (63 – 83)	67 (60 – 77)	0.05
Systolic arterial blood pressure, mmHg	100 (90 – 110)	90 (80 – 100)	0.09
Diastolic arterial blood pressure, mmHg	60 (50 – 70)	50 (50 – 60)	0.05
Arterial pH, pH unit	7.31 (7.25 – 7.37)	7.33 (7.20 – 7.40)	0.78
Bicarbonate level, mM	16.8 (14.8 – 19.2)	15.0 (11.5 – 17.8)	0.09
PCO_2 _level, Torr	34.5 (27 – 40.3)	29 (25 – 33)	0.04
Haemoglobin, g/dL	8.4 (6.4 – 9.9)	8.1 (4.9 – 9.7)	0.64
White Blood Cell count, 10^3^/mm^3^	12.3 (9.3 – 16.8)	13.8 (10.4 – 22.7)	0.24
Platelet count, 10^3^/mm^3^	104.5 (70.5 – 149.5)	66.5 (48 – 96.5)	< 0.01
Serum creatinine, μM	62 (53 – 80)	71 (53 – 88)	0.35
Serum urea, mM	7.0 (4.3 – 10.3)	8.6 (6.0 – 13.8)	0.03
Serum glucose, mM	5.8 (5.0 – 8.1)	3.3 (1.1 – 6.6)	< 0.01
Serum sodium, mM	131 (128 – 133)	130 (125 – 135)	0.48
Parasitaemia first day > 4%	5 (15.1)	12 (24.0)	0.41
Malnutrition*	17 (51.5)	14 (28.0)	0.03
Community-acquired infections	4 (12.1)	14 (28.0)	0.10
Quinine loading dose**	2 (6.1)	10 (20.0)	0.11
Sedation with clonazepam or thiopental	22 (66.6)	12 (24.0)	< 0.001
Blood transfusion, n (%)	18 (54.5)	21 (42.0)	0.37
Critical care complications***	15 (45.5)	16 (32.0)	0.21

Data are numbers and percentages, or medians and interquartile range (Q_1_–Q_3 _or IQR).

* NCHS Growth charts definition, 1976; ** 17 mg/kg over 4 hrs; *** nosocomial infections (pneumonia, bacteriaemia), pulmonary oedema and sub-glottic stenosis.

### Indications for endotracheal intubation and severity of malaria

Indications for mechanical ventilation were deep coma (GCS ≤ 7, n = 16), clinically evident status epilepticus or decorticate posturing (n = 20), decerebrate posturing (n = 15), shock (n = 15), cardiac arrest (n = 13), and severe respiratory distress with a PaO2/Fio2 < 300 Torr [40.1 kPa] (n = 4). Median PRISM_h24 _score was statistically lower when testing deep coma against other indications (10 *vs *15, p < 0.001), ditto for PELOD_h24 _score (2.5 *vs *13, p = 0.02). Among CM indications, PELOD_h24 _scores correlated with the degree of severity in CNS involvement (from 2.5 for deep comas to 21 for decerebrate posturing, p = 0.02).

### Mechanical ventilation, survival and predictors of fatal outcome

**Figure 1:**
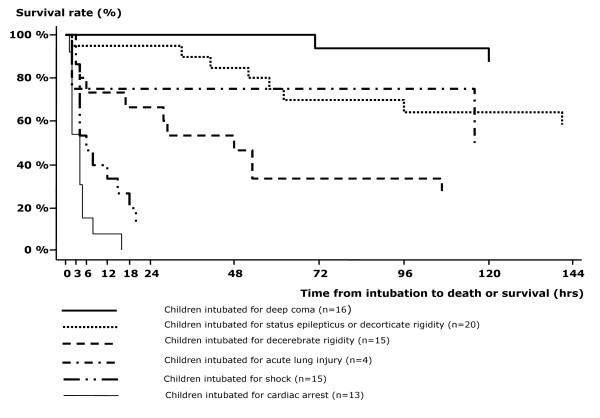
Kaplan-Meier curves related to motives of intubation among 83 children requiring endotracheal intubation for severe malaria in Dakar, Senegal.

All the children intubated for a cardiac arrest deceased within 24 hours of ventilation (Figure 1) and were subsequently excluded from the analysis. The crude and adjusted relative risks for death for the motives of intubation and other predictors are displayed in Table 2. Decerebrate posturing and shock were associated with an increased risk of death compared to deep comas. Acidosis (bicarbonate level < 15 mmol/L or bass excess <-8) was not associated with fatal outcome (p = 0.09) but there was a discrepancy in blood gases realization, performed in 97% of survivors but only in 74% of non-survivors. Thrombocytopaenia and hypoglycaemia were associated with increased mortality, whereas malnutrition and second line anticonvulsivants use were associated with survival. Lethality rates were lower with multiple convulsions (> 1/24 hrs) than with a single or no convulsion (33.0% *vs *70.2%, crude relative risk 0.5, 95% CI 0.3–0.8, p < 0.01).

**Table 2:** Relative risks for death for the motives of intubation and other predictors among children requiring endotracheal intubation for severe *falciparum *malaria in Dakar, Senegal

Indication/Variable	Patients No	Deaths (lethality %)	Crude Relative Risk ^§^	95% CI	P value	Adjusted Relative Risk ^§^	95% CI	P value
Deep coma ^a^	16	2 (12.5)	1			1		
Status epilepticus or decorticate rigidity ^b^	20	8 (40.0)	3.2	0.8 – 13.0	0.07	5.7	1.2 – 27.1	0.03
Decerebrate rigidity ^c^	15	11 (73.3)	5.9	1.5 – 22.2	<0.001	10.7	2.3 – 49.5	< 0.01
Acute lung injury ^d^	4	2 (50.0)	4.0	0.8 – 20.3	0.16	3.6	0.5 – 25.8	0.20
Shock ^e^	15	14 (93.3)	7.5	2.0 – 27.5	<0.001	19.3	4.3 – 87.5	<0.001
Thrombocytopaenia								
≥ 100,000/mm^3^	26	9 (34.6)	1			1		
< 100,000/mm^3^	42	27 (64.3)	1.9	1.0 – 3.3	0.01	2.6	1.2 – 5.8	0.01
Second line anticonvulsants ^f^								
No	36	25 (69.4)	1			1		
Yes	34	12 (35.3)	0.5	0.3 – .8	< 0.01	0.4	0.2 – 0.9	0.02
Respiratory distress ^g^								
No	26	11 (42.3)	1					
Yes	44	26 (59.1)	1.4	0.8 – 2.3	0.17			
Jaundice ^h^								
No	48	22 (45.8)	1					
Yes	22	15 (68.2)	1.5	1.0 – 2.3	0.08			
Hypoglycaemia ^i§^								
No	51	22 (43.1)	1					
Yes	19	15 (78.9)	1.8	1.2 – 2.7	< 0.01			
Severe anaemia ^j^								
No	55	26 (47.3)	1					
Yes	15	11 (73.3)	1.5	1.0 – 2.3	0.07			
Abnormal bleeding ^h^								
No	62	30 (48.4)	1					
Yes	8	7 (87.5)	1.8	1.2 – 2.6	0.06			
Renal failure ^k^								
No	68	35 (51.5)	1					
Yes	2	2 (100)	1.9	1.5 – 2.5	0.49			
Community-acquired infections ^l^								
No	58	29 (50.0)	1					
Yes	12	8 (66.6)	1.3	0.8 – 2.1	0.29			
Malnutrition ^m^								
No	44	28 (63.6)	1					
Yes	26	9 (34.6)	0.5	0.3 – 1.0	0.02			
Severe hyponatremia ^n^								
No	62	31 (50.0)	1					
Yes	8	6 (75.0)	1.5	0.9 – 2.4	0.18			

Definitions : ^a ^deep coma : Glasgow coma score (GCS) ≤ 7 or Blantyre coma score (BCS) ≤ 2, no clinically evident status epilepticus, no abnormal posture ; ^b ^coma with overt cortical/diencephalic injury as witnessed by persistent clinically evident status epilepticus or flexor (decorticate) posturing; ^c ^coma with brainstem involvement as witnessed by extensor or opisthotonic (decerebrate) posturing; ^d ^severe respiratory distress with pulmonary infiltrates on X-ray with a ratio PaO2/FiO2 < 300 Torr [40.1 kPa]; ^e ^shock defined as a systolic blood pressure <60 or <80 mm Hg in children < than or > than 5 five years, respectively, in addition to the presence of perfusion abnormalities (urine output < 1 ml/kg/hr, GCS < 15, delayed capillary refill);^f ^continuous infusions of clonazepam or thiopenthal;^g ^sustained low chest wall recession or deep acidotic breathing; ^h ^clinical criterion; ^i ^glucose level < 2.2 mmol/L; ^j ^haemoglobin rate < 5 g/dL; ^k ^abnormal ratio creatinine/age; ^l ^septicaemia, pneumonia, meningitis or dysenteric; ^m ^NCHS Growth charts definition, 1976; ^n ^sodium<125 mmo/L

^§ ^For bivariate and multivariate analysis (n = 70), children with cardiac arrests (n = 13) and children without platelet counts (n = 2) were excluded.

The Cox model showed deep coma was the indication of better outcome than other situations (fatality rate, 12.5% *vs *40 to 100%, p < 0.0001) and sedation remained significantly associated with survival. Thrombocytopaenia was an independent predictor for death. Conversely, in children with MODs, platelet counts correlated to the amount of ODs (from 90,000 with two ODs to 35,000 with four or more, p < 0.001) and platelet counts under 50,000/mm3 were only observed in children with MOD syndrome.

### Mechanical ventilation and functional outcome

Of the survivors, neurological status on discharge was normal in 30% (n = 10) or impaired in 70% (n = 23), and median PCPC score was 2 (IQR: 1–3). Compared to deep coma, PCPC score was significantly higher in other motives for intubation (6 *vs *2, p < 0.001). Only three severe disabilities (two total dependences and one vegetative state) persisted after a 2-month follow-up, occurred in two children intubated for opisthotonus, and in another one intubated for an acute lung injury.

### Influence of complications on outcome

**Figure 2:**
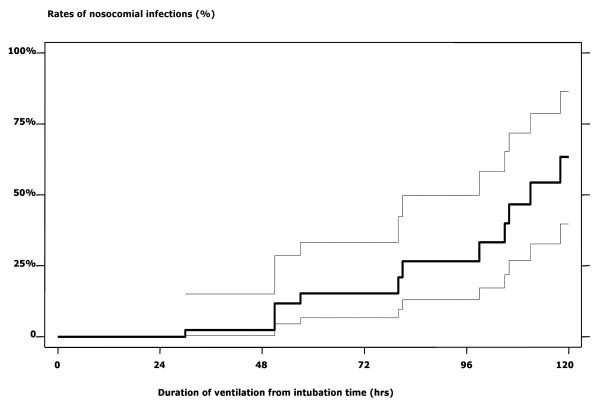
Kaplan-Meier curves and 95% confidence interval related to the incidence of nosocomial infections* among 83 children requiring endotracheal intubation for severe malaria in Dakar, Senegal. * Pneumonia (n = 12), and septicaemia (n = 6) with Staphylococcus coagulase negative (n = 2), Enterobacter sp. (n = 2), Acinetobacter sp. (n = 1), Pseudomonas sp. (n = 1)

Of 18 (21.7%) patients with community-acquired infections, no significant difference was noted between survivors and non-survivors. Among the 61 children surviving more than two days, 20 (32.8%) experienced 23 episodes of hospital-acquired infections. Cumulative incidence, as correlated with duration of ventilation, was as follows: 2.3% after 48 hrs, 15.3% after 72 hrs, 26.6% after 92 hrs, 63.4% after 120 hrs (Figure 2).

Nine children developed pulmonary oedema with ALI, 4 of which evolved to fatal ARDS. Three children required haemodialysis, none survived. Three children had an inspiratory dyspnea after extubation. Two improved after reintubation, but the third died (he was found dead at day 23 without explanation). However, the survival and functional outcome with these complications were not affected after controlling for the indications for intubation.

## Discussion

This article is the first to report a series of African children living in an hypoendemic area, treated with mechanical ventilation for life-threatening falciparum malaria. For ethical reasons, the study involved no control group, *i.e.*, non-ventilated group, since the Hôpital Principal had ventilation capability for years. Benchmarking of other CM series was not possible, since CM cases were not ventilated and had different epidemiological (*e.g.*, level of transmission or age) or clinical characteristics (*e.g.*, criterion for deep coma). This could lead to misleading interpretations of severity and fatality rates [2,3,21,23-26,28]. In this series, the high lethality could be explained by the extreme severity of our cases, as reflected by the high frequency of MOD syndrome (91%), indisputable indications for resuscitation, and PRISM score in ventilated children (median PRISM_h24_: 14, IQR: 10–19) twice as high as in our hospital's overall SM cases at the same period (median PRISM_h24_: 7, IQR: 3–13), of which fatality rate was 12% [29,30]. However, the lethality in ventilated children was close to those seen in adults with malaria treated in ICU's [14-17,31].

Acidosis, a major contributor for death, both in adults and in children [12,13,16,17], was not associated with fatal outcome when measured at admission in ventilated children, but the analysis was underpowered by missing data which might have led to underestimate severe acidosis in high-risk children.

CM was the principal condition accounting for more than 80% of our critically-ill SM cases. This is consistent with hospital-based studies highlighting CM as the most frequently fatal clinical form in hypoendemic settings [4,9]. Among neurological signs, decerebrate rigidity was associated with a far worse prognosis than decorticate rigidity. Brainstem signs, have been shown to predict a poor outcome [3,21,28]. They are associated with different underlying mechanisms, among which intracranial hypertension is the cornerstone [32,33]. Indeed, in a recent study by the KEMRI, Idro et al. have found an increased severity of the CNS involvement from decorticate posturing to opisthotonus [33]. In their study, decerebrate and opisthotonic posturing were associated with both raised intracranial pressure (ICP) and seizures, while decorticate posturing was associated only with raised ICP. The findings reported herein are consistent with these results.

Surprisingly, children with multiple convulsions had lower case fatality rates than patients with one or no convulsion. Given the lack of EEG facilities in our center, this might be explained by the failure to diagnose subtle *status epilepticus*, which would have deprived these patients of the benefit of second line anticonvlusivants, as recently described in African children [34]. This hypothesis is supported by the better prognosis associated with more efficient drugs, *e.g.*, clonazepam or thiopenthal, than in absence of treatment or when phenobarbital was the sole anticonvulsivant. Non-ventilated children with CM receiving prophylactic phenobarbital have increased mortality rates, possibly caused by drug-induced respiratory depression [35]. Mechanical ventilation or maybe just intubation for airways protection could be of potential benefit in these children.

All children intubated for shock displayed a hyperkinetic profile (rapid pulse rate without clinical sign of cardiac failure) prior to intubation. This pathophysiological profile has already been shown to predict multiple organ failure (MOF) and poor outcome in non-immune adults with SM [16,17,36]. In ventilated children, no particular organ failure predicted a fatal outcome at time of intubation. However, since most of the patients had CM and PELOD scores correlated with the degree of severity in CNS involvement, brainstem signs, *e.g.*, decerebrate/opisthotonic posturing, could be explained by the progression of MOD syndrome and CNS failure.

A platelet count under 100,000/mm^3 ^was associated with a higher risk for death. All the patients were living in and round Dakar, an hypoendemic area where the prognostic value of thrombocytopaenia in paediatric falciparum malaria had been previously reported [37]. More recently, Bruneel et al. found an increased mortality in thrombocytopaenic adults with severe imported malaria [16]. In adult ICU's, whatever the disease, the extent or the persistence of thrombocytopaenia is often a hallmark of impending death and reflects numerous mechanisms [38]. In paediatric CM, several mechanisms have been suggested, but to date only platelet sequestration in cerebral microvasculature has been associated with death [39]. In light of a previous study [37], the relationship between thrombocytopaenia and organ dysfunctions presented herein should encourage the early assessment and monitoring of platelet counts in critically-ill patients with SM.

On discharge, neurological disorders were observed in 70% of children requiring ventilation for life-threatening falciparum malaria. Nevertheless, except for three cases, these disabilities were mild to moderate and disappeared or regressed at follow-up, as previously reported [6-9,26]. However, in this retrospective study, cognitive or learning impairments could not be appraised and long-term issues were not assessed.

Another noteworthy feature was that the incidence of nosocomial infections rose from 2.3% to 63.4% after 48 hrs and 120 hrs of ventilation, respectively. Its prevalence (24.2%) was twice those reported in an Indian ICU [31], but was close to the 23–25% observed in Europe, both in PICU's and in adults referred in ICU's with a primary diagnosis of SM [17,40]. This equivalence was seen despite a poor hygienic environment in Dakar, *e.g.*, impossibility of isolating children in the two paediatric emergency rooms, and a lack of nurses. In our setting, interventions such as efforts to increase hygiene and nurse ratios might reduce the occurrence of hospital-acquired infections. Another important issue was the higher rate of ALI/ARDS in mechanically-ventilated children than in other African paediatric series [2-4,29]. This discrepancy may result both from malaria and resuscitation, as observed in adults [31,41,42]. Lastly, the high incidence of sub-glottic stenosis (3.6%) could be related to emergency intubations with non-systematic use of cuffed tubes. Nonetheless, taken together these complications had no significant influence on outcome.

There are several potential benefits of endotracheal intubation and mechanical ventilation in children with life-threatening malaria: (i) airways protection in comatose children; (ii) prevention or treatment of hypoventilation in comatose children, especially for those requiring anticonvulsive drugs; (iii) optimizing oxygenation in case of pulmonary malaria, ALI or ARDS [42,43]. This latter group will have very little chance of survival in the absence of ventilation, whether using low tidal volumes with PEEP, as performed in the Hôpital Principal, or inversion of I/E ratio or prone position, two methods not used in this hospital for technical reasons. However, the idea that mechanical ventilation may be protective for life-threatening childhood malaria had no experimental verification beyond data from comparison of patients with different complications.

## Conclusion

Finally, mechanical ventilation for resuscitation of life-threatening childhood malaria was required for a short time and its outcome depended more on clinical presentation than on critical care complications. Despite the retrospective uncontrolled design of this study, it is thought that in the absence of such technical support, more patients would have died. Then, the better outcome in deep comas would support early intubation for airways protection in CM, *e.g.*, as soon as GCS falls to 7 (or BCS falls to 2), and before progression towards MOF or brainstem involvement, two intimately tied complications difficult to cure in low-standard-care countries.

Effective paediatric intensive care for severe childhood malaria exists in a sub-Saharan African country. Further investigations are warranted to ascertain whether endotracheal intubation is effective and if so, whether the capability for it should be more widely developed in malaria endemic areas.

## Competing interests

The author(s) declare that they have no competing interests.

## Authors' contributions

P.G., A.S.K., P.J., B.D. and P.I. took part in the clinical management of cases and did the data collection. P.I and P.G designed the study, reviewed the data for inconsistencies and entry errors prior to computerization into the database. C.R and P.G. performed the statistical analysis. P.G., P.I. and C.R. wrote the manuscript which was critically reviewed by all authors. This study was not supported by funding research.
